# “Turn on” Fluorescence Sensor of Glutathione Based on Inner Filter Effect of Co-Doped Carbon Dot/Gold Nanoparticle Composites

**DOI:** 10.3390/ijms23010190

**Published:** 2021-12-24

**Authors:** Thi-Hoa Le, Ji-Hyeon Kim, Sang-Joon Park

**Affiliations:** Department of Chemical and Biological Engineering, Gachon University, Seongnam 13120, Korea; hoale2907@gmail.com (T.-H.L.); jihyeon@gachon.ac.kr (J.-H.K.)

**Keywords:** glutathione, NPCD–AuNP composites, inner filter effect (IFE), “turn on” fluorescence

## Abstract

Glutathione (GSH) is a thiol that plays a significant role in nutrient metabolism, antioxidant defense and the regulation of cellular events. GSH deficiency is related to variety of diseases, so it is useful to develop novel approaches for GSH evaluation and detection. In this study we used nitrogen and phosphorus co-doped carbon dot-gold nanoparticle (NPCD–AuNP) composites to fabricate a simple and selective fluorescence sensor for GSH detection. We employed the reductant potential of the nitrogen and phosphorus co-doped carbon dots (NPCDs) themselves to form AuNPs, and subsequently NPCD–AuNP composites from Au^3+^. The composites were characterized by using a range of spectroscopic and electron microscopic techniques, including electrophoretic light scattering and X-ray diffraction. The overlap of the fluorescence emission spectrum of NPCDs and the absorption spectrum of AuNPs resulted in an effective inner filter effect (IFE) in the composite material, leading to a quenching of the fluorescence intensity. In the presence of GSH, the fluorescence intensity of the composite was recovered, which increased proportionally to increasing the GSH concentration. In addition, our GSH sensing method showed good selectivity and sensing potential in human serum with a limit of detection of 0.1 µM and acceptable results.

## 1. Introduction

Glutathione (GSH) is a tripeptide compound produced by the liver that is composed of three different amino acids: cysteine, glutamic acid and glycine (ƴ-Glu-Cys-Gly) [1]. GSH acts as an antioxidant and is involved in many processes in the body, including tissue building and repair, cysteine transport and storage, signal transduction modulation, cell propagation control and immune system response regulation [2,3]. Abnormal levels of GSH have been reported to contribute to oxidative stress, which plays a key role in aging, and in a variety of diseases, including cancer, neurotoxicity, psoriasis, liver damage, Parkinson’s disease, and Alzheimer’s disease. Therefore, GSH is considered a universal biomarker in the diagnosis and therapeutic monitoring of cancers [4,5].

Many techniques have been used for the identification and quantification of GSH, such as HPLC [6], electrochemistry [7], fluorescence [8], electrochemiluminescence [9] and surface enhanced Raman scattering (SERS) [10].

Fluorescence-based methods are widely used for sensing different analytes based on photoinduced electron transfer (PET), photoinduced charge transfer (PCT), fluorescence resonance energy transfer (FRET) and the inner filter effect (IFE). PET and PCT are intramolecular processes, whereas IFE and FRET are intermolecular processes, all of which involve energy transfer between at least two independent molecules [11,12,13,14]. We developed FRET-based sensors in previous studies and designed detection methods that reply via IFE in this study. The IFE is a decrease in the fluorescence intensity of a fluorophore (donor) due to the absorption of the excitation/emission light by an absorber (acceptor). This occurs when the absorption spectrum of the absorber overlaps with the fluorescence excitation or emission spectrum of the fluorophore [14]. The IFE is classified as primary when the excitation beam is attenuated by the volume of the sample, and secondary when the emission of the fluorophore is absorbed by a molecule present in the solution [15]. Unlike FRET-based sensors, IFE-based sensors do not provide site-specific information and cannot be used to determine donor–acceptor interactions. However, the IFE has been shown to be useful for converting analytical absorption signals into fluorescence signals, which has been proven to enhance sensitivity and selectivity [16,17]. Moreover, IFE-based sensors do not require any surface modification, or covalent linking between a donor and acceptor, which makes probe fabrication simpler and flexible [18]. 

Recently, the combination of carbon dots (CDs) and gold nanoparticles (AuNPs) has been shown to be an effective IFE pair because of the outstanding properties of both materials. CDs are a promising class of fluorescent carbon materials that have attracted considerable interest because of their distinctive properties, such as excellent solubility, low toxicity, good biocompatibility, high photostability, tunable surface functionalities and cell membrane permeability [19,20]. AuNPs have been widely used in fundamental studies for a variety of applications. It is worth noting that the AuNP solutions have different colors depending on their size distributions and morphologies, which is advantageous for the recognition of different target molecules [21,22,23]. In addition, AuNPs are ideal fluorescent absorbers because of their high extinction coefficients, and extensive absorption spectrum that can easily overlap with the excitation/emission spectra of CDs to produce an IFE process [24,25]. 

Many previous studies have used CD/AuNP pair-based IFE mechanisms for sensing analytes, such as hyaluronidase [26], aldicarb [27], metformin hydrochloride [28], cyanide ion [29] and biothiols. For the analytes, AuNPs were obtained via the reduction of chloroauric acid using tri-sodium citrate as a reductant and surfactant. This results in several negatively charged citrate ions on the surfaces of AuNPs, which may affect the IFE process. Most fluorescence-based sensors have focused on the fluorescence properties of CDs. However, it is beneficial to take full advantage of CDs, including their reducing and stabilizing potential. CDs synthesized using the hydrothermal method contain a large number of carboxyl and hydroxyl groups [30]. Negatively charged carboxyl groups on the CDs can stabilize metal particles in solution [31]. In addition, the hydroxyl groups allow CDs to act as green reducing agents in the synthesis of metal nanoparticles [32]. Hence, the CDs have two important roles: (1) a fluorescence source, and (2) a reducing agent and stabilizer for AuNPs in the fabrication of CD/AuNP pair-based IFE sensors.

**Scheme 1:**
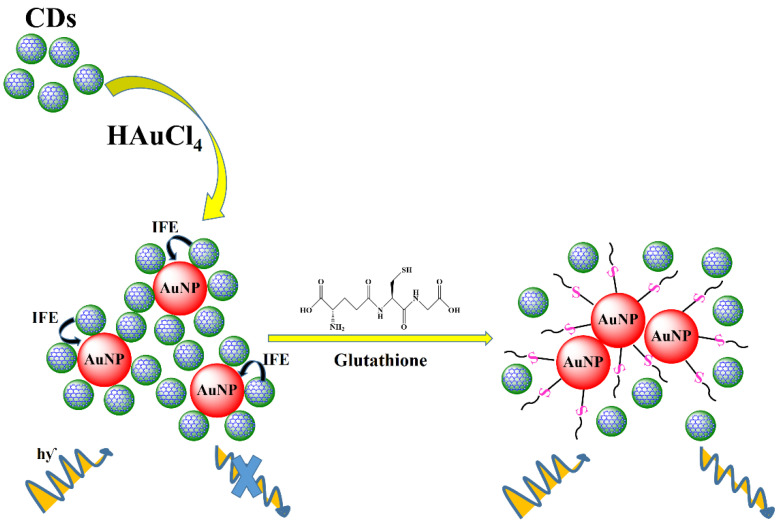
Demonstration of the glutathione detection based on inner filter effect happening in NPCD–AuNP composite material.

Another novelty of this research is the use of nitrogen and phosphorus co-doped carbon dots (NPCDs) in an IFE-based sensor for GSH detection. NPCDs possess enhanced fluorescence properties and more negatively charged functional groups and amine groups on the surface compared to pristine CDs [19]. The amine and thiol groups show strong binding affinity to the surfaces of AuNPs [33,34], which can increase the effectiveness of the IFE process between NPCDs and AuNPs. NPCDs can successfully reduce HAuCl_4_ salt to AuNPs without adding any other reductant and surfactant. Once AuNPs are formed, NPCDs act as fluorescence donors that trigger the IFE process with AuNP acceptors, which leads to the quenching of the fluorescence of NPCDs. The fluorescence of NPCDs is “turned on” after GSH is added. The introduction of GSH to the AuNP system can induce aggregation of AuNPs [24]. In addition, because of its specific multidentate and steric structure, GSH molecules are able to preferentially enclose AuNPs and isolate them from NPCDs. Hence, the interaction between NPCDs and AuNPs can be disrupted by GSH, leading to a decrease in the IFE and a restoration in the fluorescence of NPCDs. The mechanism of “turn on” fluorescence for detecting GSH is illustrated in Scheme 1. Compared to “turn off” fluorescence sensors, the “turn on” fluorescence sensors are preferable because the likelihood of false positives is reduced. 

## 2. Chemicals and Experiments

### 2.1. Chemicals

All chemicals including (NH_4_)_2_HPO_4_, citric acid monohydrate, HAuCl_4_·3H_2_O, glutathione, cysteine, homocysteine, alanine, glycine, lysine, tryptophan, methionine, glutamic acid, human serum and deionized (DI) water were obtained from Sigma-Aldrich.

### 2.2. Instruments

Photoluminescence (PL) and UV–Vis spectra were obtained using a QuantaMaster TM 50 PTI spectrofluorometer (Photon Technology International, San Diego, CA, USA) and a G1103A UV–Vis spectrophotometer (Agilent, Santa Clara, CA, USA). Size distribution measurements were performed using electrophoretic light scattering (ELS; Photal Otsuka Electronics, ELS 8000, Osaka, Japan). The structural and morphological characterization of the samples was carried out by scanning electron microscopy (SEM; S-4700, Hitachi Ltd., Tokyo, Japan) and transmission electron microscopy (TEM; Tecnai, F30S-Twin, Hillsboro, OR, USA). X-ray photoelectron spectroscopy (XPS) and powder X-ray diffraction (XRD) were performed using an X-ray photoelectron spectrometer (PHI 5000, Chigasaki, Kanagawa Prefecture, Japan) and X-ray source (Rigaku/Smartlab, Tokyo, Japan), respectively. 

### 2.3. Preparation of NPCDs

NPCDs were fabricated according to the method presented in our previous work [19]. In particular, (NH_4_)_2_HPO_4_ (5 g) and citric acid (2 g) monohydrate (molar ratio of 4:1) were mixed with DI water (30 mL). Subsequently, the mixture was transferred into a 50 mL Teflon-lined stainless-steel autoclave and heated at 180 °C for 4 h. After cooling, the solution was purified by filtering through a 0.22-µm polyethersulfone membrane into a dialysis bag (MWCO: 1000 Da) to dialyze for 48 h. The obtained light-violet colored solution was lyophilized to collect a powdery substance.

### 2.4. Synthesis of NPCD–AuNP Composites

The NPCD solution (1.2 mL, 0.075 g/mL) was added to DI water (10 mL). The solution was heated to boiling before 1% HAuCl_4_ (100 µL) was dropped into the solution. The color of the solution quickly changed to purple and then burgundy after 10 min, indicating the successful formation of AuNPs. 

### 2.5. Fluorescence Sensing of GSH

GSH sensing was carried out at room temperature. First, NPCD–AuNP solution (300 µL) was added to DI water (2700 µL) to obtain a solution concentration of 9.05 × 10^−4^ g/mL. Subsequently, different volumes of GSH solution were added via dropping into the NPCD–AuNP solution to obtain various concentrations from 0.1 to 300 µM. The solutions were further diluted to the mark using DI water and kept in equilibrium for 60 min before fluorescence measurements were made with an excitation wavelength of 340 nm. Each concentration was measured three times to determine the standard deviation.

### 2.6. Detection of GSH in Human Serum

Human serum (30 µL) was added to DI water (2670 µL) followed by NPCD–AuNP solution (300 µL). A quantity of GSH was dropped into the solution, followed by dilution to the mark with DI water before fluorescence analysis without pretreatment. The sensing of GSH in human serum samples was performed using the method described above.

### 2.7. Selectivity

To study the GSH specificity of the NPCD–AuNP-based fluorescence sensing approach, the fluorescence responses of other biothiols, including HCys, Cys and some amino acids, were investigated. 

## 3. Results and Discussion

### 3.1. Characterization of NPCDs-AuNPs Composites

**Figure 1:**
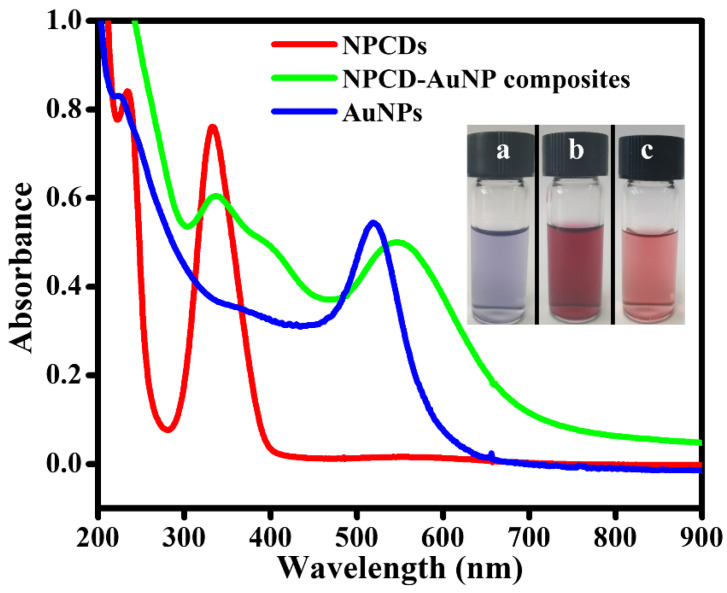
UV–Vis spectra of NPCDs, NPCD–AuNP composites and AuNPs (inset: aqueous solution of NPCDs (**a**), NPCD–AuNP composites (**b**) and AuNPs (**c**)).

UV–Vis absorption and fluorescence spectroscopy were used to characterize the optical properties of the NPCDs and NPCD–AuNP composites. The UV–Vis spectrum of the aqueous NPCD solution (Figure 1) shows a peak appears at 234 nm and another at 334 nm, which originate from the surface states [20,35,36]. There is a peak located at 521 nm in the spectrum of AuNP solution synthesized using sodium citrate as a reductant. The maximum absorption peak of AuNPs can redshift with an increase in size of AuNPs [37]. The spectrum of an aqueous solution of NPCD–AuNP composites shows the same peak of NPCDs at 334 nm and a specific peak at 548 nm, which confirms the successful reduction of NPCDs to form AuNPs.

**Figure 2:**
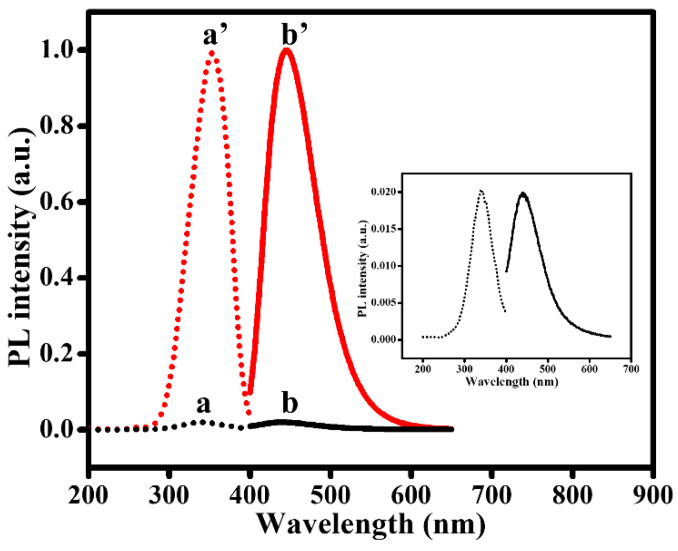
Excitation (a, a’) and emission (b, b’) fluorescence spectra of the NPCDs (a’, b’) and NPCD–AuNP composites (a, b) (inset: enlarged fluorescence spectrum of NPCD–AuNP composites).

The NPCD and NPCD–AuNP composites’ fluorescence emission peaks appeared at 447 and 442 nm under excitation wavelengths of 355 and 340 nm, respectively (Figure 2). Therefore, there was a blue shift in the emission and excitation peaks for the NPCD–AuNP composites. In comparison to the fluorescence intensity of the NPCDs, that of the NPCD–AuNP composites was considerably quenched. As shown in Figure 1, the absorption spectrum of the NPCD–AuNP composites presents a broad peak between 440 and 650 nm with a peak maximum at 548 nm because of the presence of AuNP components, and the emission peak is located at 442 nm due to the NPCD components. Hence, an overlap between the absorption and emission peaks in the composite material easily produced an IFE process, leading to the quenching of fluorescence intensity.

**Figure 3:**
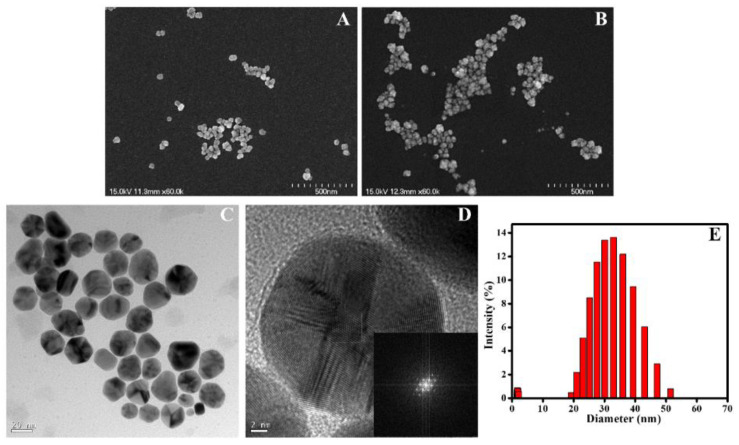
SEM images of NPCD–AuNP composites in the (**A**) absence and (**B**) presence of GSH. (**C**) TEM image. (**D**) High-resolution TEM image (inset: fast Fourier transform (FFT) pattern). (**E**) DLS analysis of NPCD–AuNP composites.

The morphology and size of the NPCD–AuNP composites were analyzed by SEM, TEM and DLS. Under the influence of NPCDs, the synthesized AuNPs were relatively monodispersed and spherical. They had a narrow size distribution range of 19.2 to 51.5 nm and an average size of 32.5 nm (Figure 3). A high-resolution (HR) TEM image (inset of Figure 3D) shows a crystal of the AuNPs, which was further characterized by powder XRD. 

**Figure 4:**
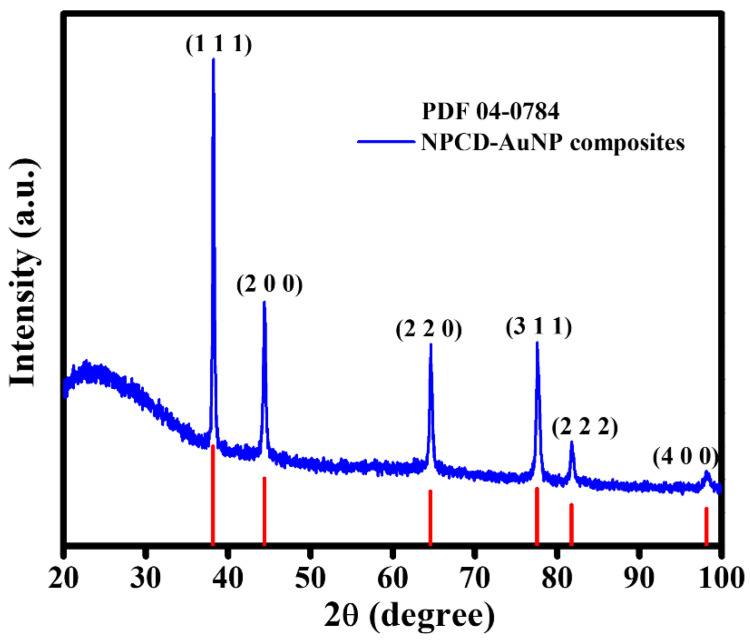
Powder XRD pattern of NPCD–AuNP composites.

The powder XRD pattern of the NPCD–AuNP composites is shown in Figure 4. The composites exhibited six planes of (111), (200), (220), (311), (222) and (400), which were assigned to sharp peaks at 38.2°, 44.4°, 64.5°, 77.5°, 81.7° and 98.1° and lattice fringe distances of 2.36, 2.04, 1.44, 1.23, 1.18 and 1.02 Å, respectively. Hence, the pattern closely matches with the XRD pattern of pure crystalline Au° with face-centered cubic crystal structures (PDF 04-0784).

**Figure 5:**
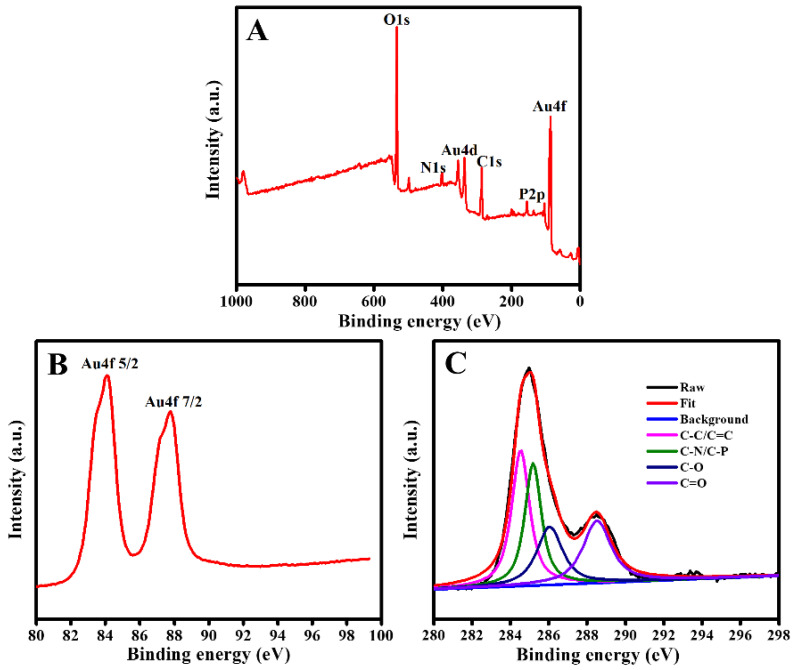
(**A**) XPS survey spectrum of NPCD–AuNP composites. High resolution XPS spectra of Au4f (**B**) and C1s (**C**).

The XPS profile (Figure 5A) presents typical peaks at 83.97, 133.71, 284.92, 336.14, 354.3, 400.5 and 532.33 eV, corresponding to Au4f, P2p, C1s, Au4d (Au4d_5/2_ and Au4d_3/2_), N1s and O1s, respectively. The atomic percentages of Au, C, O, N and P were found to be 5.35%, 39.18%, 45.61%, 6.98% and 2.87%, respectively. The high-resolution XPS profile of the Au4f peak is shown in Figure 5B. Two peaks of Au4f_5/2_ and Au4f_7/2_ located at 84.4 and 87.7 eV, respectively, correspond to the metallic state of Au^0^. Figure 5C shows that the C1s peak can be parsed to four peaks at 284.2, 285, 286 and 288.4 eV, which are assigned as C-C/C=C, C-N/C-P, C-O and C=O bonds, respectively. These peaks originate from the sp^2^ graphitic structure [38] and several carboxyl, hydroxyl, amine and phosphate groups on the surfaces of the NPCDs. These assignments can be further confirmed from the P2p and N1s spectra, which are similar to those measured in the our previous study [19]. 

### 3.2. Detection of GSH

#### 3.2.1. Mechanism of Sensing

**Figure 6:**
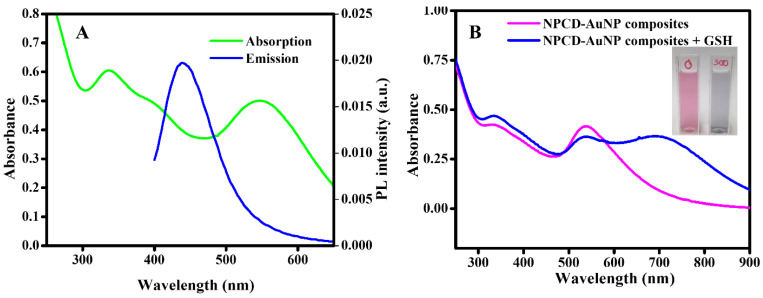
(**A**) Overlap of absorption peaks and emission peaks of NPCD–AuNP composites. (**B**) Absorption spectra (inset: image of colored solution) of NPCD–AuNP composites in the presence and absence of GSH (300 µM).

**Figure 7:**
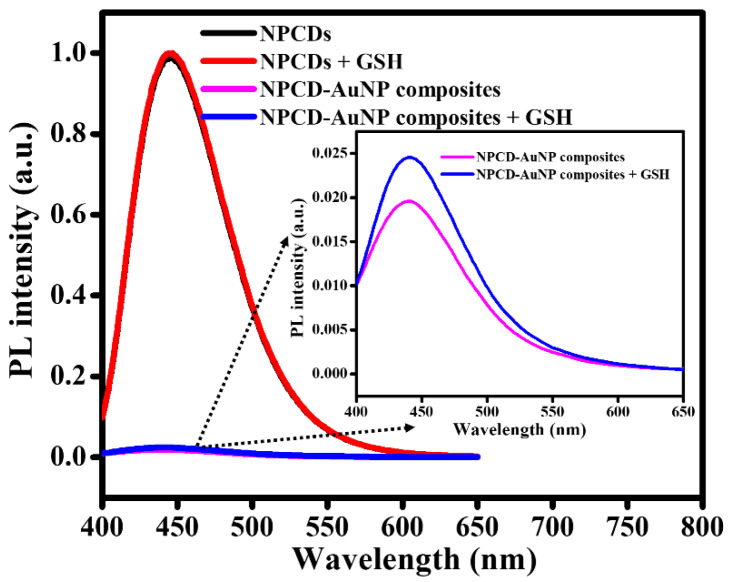
Variation in fluorescence intensity of NPCDs and NPCD–AuNP composites (inset: enlarged fluorescence spectra of NPCD–AuNP composites) in the presence and absence of GSH (40 µM).

As previously discussed, the overlap of the absorption spectrum of the AuNP components and the emission spectrum of the NPCD components led to a significant quenching in the fluorescence intensity of the NPCD–AuNP composites compared to that of pure NPCDs. This overlap is illustrated in Figure 6A. However, the intensity of the NPCD–AuNP composites recovered in the presence of GSH. This phenomenon did not occur with NPCDs. In other words, GSH cannot affect the fluorescence of NPCDs but can turn on that of the NPCD–AuNP composites (Figure 7). This is explained by the fact that GSH can bind to AuNP surfaces via Au–S bonding and subsequently induce the aggregation of AuNPs [34,39]. This can be clearly seen in the SEM images of the NPCD–AuNP composites in the presence and absence of GSH (Figure 3A,B). Moreover, the color of the composite solution changed from red to purple blue, and the absorbance peak of the composites redshifted (Figure 6B), further indicating the aggregation of AuNPs in the composites after the addition of GSH. Aggregation may limit the ability of AuNPs to act as acceptors in the IFE process. Furthermore, GSH shows a strong affinity to bind AuNPs through its multidentate ligands, and has a steric structure that can keep the bound AuNPs separate from the NPCDs, which results in fluorescence recovery of NPCDs. 

**Figure 8:**
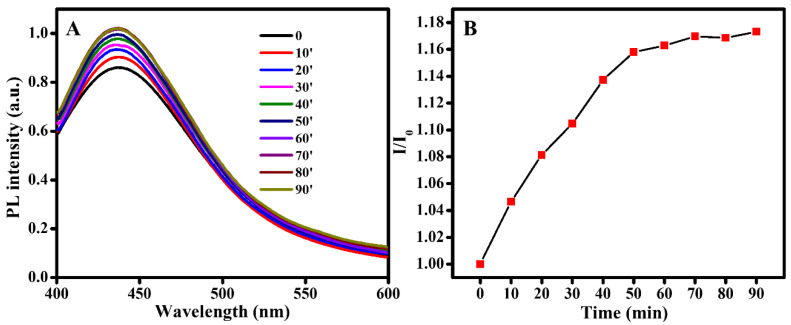
(**A**) Fluorescence intensity of NPCD–AuNP composites with GSH over time. (**B**) Relative fluorescence (I/I_0_) of NPCD–AuNP composites over time with 20 µM GSH.

After adding GSH to a fixed concentration of NPCD–AuNP composites, the fluorescence intensity was recorded every 10 min to investigate the kinetics of the IFE process. As shown in Figure 8, the intensity of the composites increased steadily in the presence of GSH from 0 to 60 min. Thereafter, the intensity remained relatively stable. Therefore, 60 min was chosen as the optimal time for the next step.

#### 3.2.2. GSH Sensing

**Figure 9:**
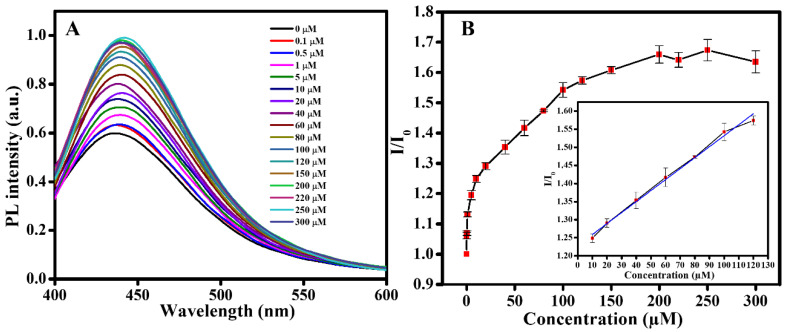
(**A**) Fluorescence spectra. (**B**) Relative fluorescence of NPCD–AuNP composites in the presence of various concentrations of GSH (inset: linear relationship between I/I_0_ and GSH concentration in the range of 10 to 120 µM).

Figure 9 clearly shows the fluorescence recovery of the NPCD–AuNP composites in the presence of GSH. The fluorescence intensity of the composites increased steadily when the GSH concentration was increased from 0 to 300 µM. When the GSH level was within the range of 10–120 µM, there was a linear relationship between the NPCD–AuNP composite intensity and the GSH concentration, I/I_0_ = 1.22872 + 0.00303C_M_, with a correlation coefficient (R^2^) value of 0.99716. The measurement was performed three times, and the data were plotted with standard deviations. In addition, the limit of detection (LOD) value was determined to be approximately 0.1 µM. A comparison of the detection results using our approach with those of other approaches is presented in Table 1.

#### 3.2.3. Sensing in Serum

To further investigate the practicality and reliability of the NPCD–AuNP composite-based sensor, we tested this material for GSH detection in human serum. The results are displayed in Table 2. The recovery range of GSH was 90.74–107.04%. The experiment was repeated three times, and all relative standard deviations (RSDs) were less than 5%. These results indicate that the method has potential applications for practical the detection of GSH. 

#### 3.2.4. Selectivity

**Figure 10:**
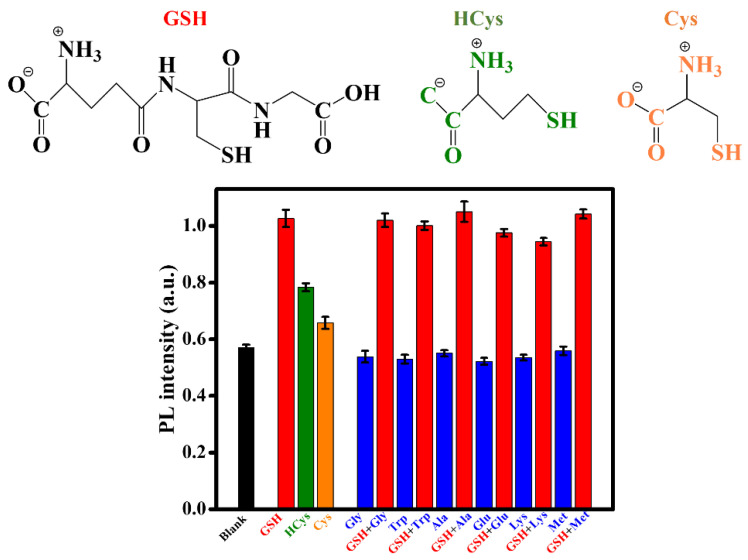
Fluorescence response of NPCD–AuNP composites on adding 200 µM of different biothiols and amino acids.

High specificity is a necessary condition for a good sensor. To confirm that NPCD–AuNP composites are specific to sensing GSH, the “turn on” fluorescence responses of other biothiols—including Cys; HCys; and amino acids, such as glycine (Gly), alanine (Ala), lycine (Lys), trytophan (Trp), glutamic acid (Glu) and methionine (Met)—were studied in the absence and presence of GSH. As shown in Figure 10, all biothiols, such as GSH, Cys and Hcys, enhanced the fluorescence intensity of the composites. On the other hand, the addition of an amino acid did not increase the fluorescence intensity of the composites or affect the role of the fluorescence “turn on” of GSH. The introduction of biothiols to the fluorescent NPCD–AuNP composites can induce the aggregation of AuNPs in the order of HCys >> Cys > GSH [48]. The intensity was the strongest in the presence of GSH, followed by HCys and Cys. This is explained by the high denticity of GSH, which is typically two or more parts of the molecule (–SH and –COO^−^). This chelation makes the GSH–metal atom interactions stronger than interactions of metal atoms with the other two biothiols or amino acids that possess either a single –SH, weak amine or –COO^−^ binding group [49]. In addition, the large steric hindrance effect created by GSH is greater than that of Cys or HCys, so it is much more able to coordinate stably to the metal atoms or ions [50]. In addition, many studies indicate that GSH is superior to Cys and HCys in the role of a capping ligand for semiconductor nanocrystals [51]. For these reasons, the interaction between GSH and AuNPs is much stronger than the interactions of the other molecules studied. This results in significant enhancement in the fluorescence intensity of the composites only when GSH is added, which ensures that the sensor based on NPCD–AuNP composite material is highly selective for GSH sensing.

## 4. Conclusions

In summary, we successfully synthesized NPCD–AuNP composites, which are ideal IFE pairs for GSH sensing applications. We effectively utilized the enhanced fluorescence properties of NPCDs and their reducing nature to synthesize AuNPs and the subsequent composites from an Au^3+^ salt. The fluorescence of the NPCD–AuNP composites recovered steadily with increasing concentrations of GSH. A linear relationship of fluorescence against GSH concentration from 10 to 120 µM was determined, with an R^2^ value of 0.99716 and LOD value of 0.1 µM. In comparison to the results of some previous studies, our sensing results were not as sensitive. However, our NPCD–AuNP composite-based GSH sensor does not require a complicated design or expensive instruments. Its simplicity, sensitivity, selectivity and effectivity make it a potentially viable sensor for GSH detection. Thus, we have introduced a new strategy for development of simple sensors for biomedical applications.

## Figures and Tables

**Table 1:** Comparison of the GSH detection results of our method to those of other methods.

Method	Linear Range	LOD	Reference
Colorimetric		0.03 mM	[40]
Fluorescence	0.07–70 µM	48 nM	[41]
Fluorescence	0.5–300 µM	0.26 µM	[42]
Fluorescence	0–50 µM	0.19 µM	[43]
Electrochemical	10–250 µM	25 µM	[44]
Electrochemical	10 µM–500 mM	703 nM	[45]
Electrochemical	100 nM–10 mM	41.9 nM	[46]
Photoelectrochemical	1–10 µM	0.8 µM	[47]
Fluorescence	10–20 µM	0.1 µM	Our method

**Table 2:** Determination of GSH in human serum.

**Sample**	**Added (µM)**	**Founded (µM)**	**Recovery (%)**	**RSD (*n* = 3)**	
1	10	9.07	90.74	2.27	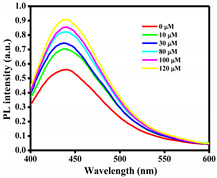
2	30	31.49	104.96	4.54	
3	80	78.19	97.73	2.75	
4	100	95.43	95.43	4.35	
5	120	128.45	107.04	1.55	
